# Mesh nebulizer is as effective as jet nebulizer in clinical practice of acute asthma in children

**DOI:** 10.3906/sag-1812-133

**Published:** 2019-08-08

**Authors:** Özge SOYER, Melike KAHVECİ, Betül BÜYÜKTİRYAKİ, Ebru ARIK YILMAZ, Betül KARAATMACA, Saliha ESENBOĞA, Pınar GÜR ÇETINKAYA, Ümit Murat ŞAHİNER, Bülent Enis ŞEKEREL

**Affiliations:** 1 Department of Pediatric Allergy, Medical Faculty, Hacettepe University, Ankara Turkey

**Keywords:** Asthma, children, jet nebulizer, mesh nebulizer, spirometry, whole body plethysmography

## Abstract

**Background/aim:**

The aim of this study was to compare the effect of salbutamol delivered to children by jet nebulizer (JN) and mesh nebulizer (MN).

**Materials and methods:**

Children admitted with acute asthma were treated with 3 doses of nebulized salbutamol, 1 given by MN. The patients’ vital signs, lung function measurements, modified pulmonary index score (MPIS), and whole body plethysmography (WBP) measurements were evaluated before and 20 min after each dose of salbutamol.

**Results:**

Thirty-one****children [9.5 (6.4–17.2) years, 67.7% male, 32.3% female] with mild (67.7%) and moderate (32.3%) asthma attacks were included in the study. The improvements with MN were comparable with JN in terms of changes in pretreatment and posttreatment forced expiratory volume in the first second (FEV1) (2.57 ± 4.57, 3.65 ± 5.44; P = 0.44), forced vital capacity (FVC) (2.52 ± 5.29, 4.17 ± 7.54; P = 0.28), heart rate (7.33 ± 10.21, 4.14 ± 9.32; P = 0.24), peripheral capillary oxygen saturation (SpO2) (0.38 ± 0.23, 0.43 ± 0.15; P = 0.83), and modified pulmonary index score (MPIS) (−6.30 ± 22.70, −8.77 ± 25.46; P = 0.70). The pre- and posttreatment values of total lung capacity (TLC), residual volume (RV), specific conductance (sGaw), and RV/TLC were similar for the JN and MN groups. Adverse effects were not different: however, complaints of palpitation were significantly higher in the posttreatment MN group than the pretreatment MN group (32.3% vs 9.7%, respectively, P = 0.016).

**Conclusions:**

These findings support the previous evidence found in studies of adults that MN is as effective as and as safe as JN in the treatment of acute asthma in children.

## 1. Introduction 

In the treatment of acute severe asthma, inhalation therapy through nebulizers is essential. Nebulizers are devices that convert liquid formulations into gaseous suspended droplets. There are 3 types of nebulizers that have different working principles currently in use: jet, ultrasonic, and mesh nebulizers. Jet nebulizers (JN) are in widespread use for the treatment of acute asthma in daily practice, since they are less expensive, less fragile, and have a smaller average particle size than ultrasonic nebulizers. However, ultrasonic nebulizers have a higher output rate and do not increase drug concentration as much as JN, but may cause drug degradation, and do not nebulize suspensions well [1]. Recently, several electronic nebulizer devices that use a vibrating mesh or plate have been marketed, and these devices have been suggested to be more efficient at delivering aerosol to the lung.Mesh nebulizers (MN) have the advantages of being quieter, lighter, portable, suitable for use in a horizontal position, and with no need for a continuous electric supply. Despite the limited number of studies reporting a comparable effect with JN, there is no consensus on which nebulizer is more appropriate for the treatment of acute asthma or which is better for certain subgroups of children [2,3].

We have therefore undertaken a randomized, single-blind clinical trial to compare the effect of salbutamol delivered by JN and MN on clinical and lung function parameters in children with mild/moderate acute asthma. The primary outcome goal was to evaluate whether nebulized salbutamol via 2 different nebulizers provides similar benefit to forced expiratory volume in the first second (FEV1). Secondary outcomes included changes to peripheral capillary oxygen saturation (SpO2), modified pulmonary index score (MPIS), forced vital capacity (FVC), FEV1/FVC, total lung capacity (TLC), residual volume (RV), RV/TLC, specific conductance (sGaw), and frequency of adverse effects (palpitation, shivering, tremor, and flushing).

## 2. Materials and methods 

This prospective study was performed in a tertiary pediatric allergy department between 2015 and 2018. The patients between the ages of 6 and 18 years who presented with mild-to-moderate acute asthma and were cooperative for performance of lung function tests (LFT) and whole body plethysmography (WBP) were included. Acute asthma was defined as an increase in symptoms, such as cough, wheezing, shortness of breath or chest tightness, and beta2-agonist use [4]. Severity of acute asthma was evaluated based on the 6 variables of the MPIS: heart rate, respiratory rate, inspiratory-to-expiratory flow ratio, accessory muscle use, degree of wheezing, and oxygen saturation in room air [5,6]. The patients who had used long-acting bronchodilators within the last 12 h or short-acting bronchodilators within the last 2 h were excluded. History of chronic disease, having the signs of respiratory failure, admission to an intensive care unit in the last year and/or hospitalization history in the last 6 months, and having a contraindication for a lung function test (LFT), such as pneumothorax, were the exclusion criteria. Hacettepe University Institutional Review Board approved the study (KA14012/685), and only patients and their parents who gave written informed consent were included. 

### 2.1. Study design 

To document whether different nebulizers have comparable efficacy and safety, mesh (Omron NE-U22-E, Kyoto, Japan) and jet (Omron NE-C28-P, Kyoto, Japan) nebulizers were compared in this participant-blinded study. The children admitted with acute asthma were treated with 3 repetitive doses of nebulized salbutamol given every 20 min. Only one of the doses was given with MN, and as either the second or third dose. Pooled analysis was performed for the second and third doses according to the type of the nebulizer. Figure represents the scheme of the study. 

The data collected on admission included demographics and asthma-specific characteristics. The patients’ vital signs, SpO2, lung function measurements, and MPIS were evaluated before and 20 min after each dose of salbutamol. Salbutamol was administered to all patients as a 0.15 mg/kg/dose (max. 5mg) every 20 min using the nebulizer with a face mask. Physicians followed the recommendations of the Global Initiative for Asthma (GINA) guidelines for acute asthma treatment [4]. 

Lung function parameters were measured at admission and 20 min after each dose of salbutamol with a spirometer (ZAN100 spirometry system, nSpire Health, Longmont, CO, USA), including FVC, FEV1, FEV1/FVC, TLC, RV, RV/TLC, sGaw, and whole body plethysmography (WBP) (SensorMedics 2130 & 6200 Autobox, SensorMedics, Anaheim, CA, USA).

**Figure:**
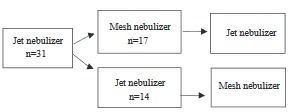
Scheme of the study.

### 2.2. Statistical analysis 

The descriptive data for categorical variables were expressed as frequencies and percentages for continuous variables and as means and standard deviations (SD) or medians and interquartile ranges, according to the distribution of variables, as appropriate. For categorical variables, the chi-square test was used to compare groups, McNemar and Cochran Q-tests were used for dependent variables. Group comparisons for variables that were not distributed normally were carried out with the Mann–Whitney U test or the Kruskal–Wallis test, and the Wilcoxon signed-ranks test and Friedman tests were used for dependent variables. A P-value of less than 0.05 was considered statistically significant. All analyses were performed using IBM SPSS Statistics 22.0 for Windows (IBM Corporation, Armonk, NY, USA). 

## 3. Results 

We included 31 children (67.7% male, 32.3% female) with a median age of 9.5 years (6.4–17.2 years). The characteristics of the patients are summarized in Table 1. Twenty-three (74.2%) patients were on prophylactic asthma medications, and 21 (67.7%) of them were using inhaled corticosteroids with a median duration of 9 months (1–36 months). At admission, 21 (67.7%) patients were classified as having mild exacerbation and the remaining (32.3%) as moderate exacerbation according to MPIS scores. Lung functions, including FEV1 and SpO2, significantly improved after 3 doses of beta2-agonist inhalation (Table 2). At admission, the mean FEV1 value was 66.66 (±17.62). The mean MPIS value at admission was 7 (± 0.39) and decreased to 5.22 (±0.41) after 3 doses of salbutamol (Table 2). Six patients (19.4%) required systemic corticosteroids after 3 doses beta2-agonist inhalation. 

**Table 1:** Characteristics of the study population (n = 31).

Male (n,%)	21 (67.7)
Age (year) *	9.5 (6.4–17.2)
Onset of asthma symptoms (year)	3.64 ± 2.89
Age of asthma diagnosis (year)	4.88 ± 3.37
Atopy n (%) Pollen Dust mites Animal dander Mold Food	18 (58)12 (38.7) 8 (25.8) 1 (3.2) 5 (16.1) 1 (3.2)
Comorbidity n (%) Allergic rhinitis Obesity Chronic sinusitis Adenoid vegetation Allergic rhinitis+obesity	15 (48.4) 10 (32.3) 2 (6.5) 1 (3.2) 1 (3.2) 1 (3.2)

*median (interquartile range)

**Table 2:** Clinical and lung function parameters of the patients at admission and after treatment.

	Admission	After 3 doses ofsalbutamol	P
FEV1 (%) predicted	66.66 ± 17.62	84.87 ± 16.28	<0.001
FVC (%) predicted	72.96 ± 17.43	87.09 ± 14.66	<0.001
FEV1/FVC	97.03 ± 14.07	102.90 ± 9.63	0.004
Sp02 (%)	96.29 ± 1.37	97.09 ± 0.97	<0.010
MPIS	7 ± 2.2	5.22 ± 2.33	<0.003
TLC (%) predicted	114.20 ± 17.78	113.76 ± 14.62	0.862
RV (%) predicted	219 ± 81.85	196.83 ± 57.98	0.105
SGaw (%) predicted	62.50 ± 38.21	113.34 ± 66.19	0.001
RV/TLC (Lt)	0.40 ± 0.11	0.36 ± 0.09	0.010

Abbreviations: FEV1, forced expiratory volume in 1 s; FVC, forced vital capacity; SpO2, peripheral capillary oxygen saturation; MPIS, modified pulmonary index score; TLC, total lung capacity; RV, residual volume; SGaw, Specific airway conductance

The MN group was comparable with the JN group in pretreatment FEV1, posttreatment FEV1, change in FEV1, pre and posttreatment FVC, FEV1/FVC, heart rate, SpO2, and pre- and posttreatment MPIS (Table 3). There was no difference in FEV1 and MPIS pre- and posttreatment in either group. The SpO2 before and after treatment was >95% in all patients and no significant changes were seen after treatment in both groups (P = 0.828, Table 4). TLC, RV, sGaw, and RV/TLC were measured by WBP and the results were similar for both groups (Table 3). Adverse effects were not different between JN and MN groups except palpitation (Table 4). Complaint of palpitation was significantly higher in the posttreatment MN group than the pretreatment MN group (32.3% vs 9.7%, respectively, P = 0.016).

**Table 3:** Comparison of mesh and jet nebulizer study groups by spirometer WBP measurements.

	Mesh nebulizer	Jet nebulizer	P
FEV1 Pre (%) (Mean ± SD) Post (%) (Mean ± SD) % Change	81.00 ± 15.1083.12 ± 16.102.57 ± 4.57	81.29 ± 16.3484.00 ± 15.833.65 ± 5.44	0.9410.8270.442
FVC Pre (%) (Mean ± SD) Post (%) (Mean ± SD) % Change	82.61 ± 15.8484.51 ± 15.972.52 ± 5.29	83.00 ± 16.1385.8 ± 14.404.17 ± 7.54	0.9250.7350.276
FEV1/FVC Pre (%) (Mean ± SD) Post (%) (Mean ± SD) % Change	103.12 ± 8.05104.12 ± 7.931.05 ± 3.63	103.67 ± 8.51103.29 ± 9.540.34 ± 5.99	0.8160.7250.217
MPIS Pre (%) (Mean ± SD) Post (%) (Mean ± SD) % Change	5.80 ± 2.455.38 ± 2.31−6.30 ± 22.70	6.00 ± 2.065.45 ± 2.41−8.77 ± 25.46	0.7330.9230.706
Heart Rate Pre (%) (Mean ± SD) Post (%) (Mean ± SD) % Change	110.00 ± 17.33118.03 ± 21.177.33 ± 10.21	111.87 ± 18.92116.22 ± 20.414.14 ± 9.32	0.7090.7610.247
Sp02 Pre(%) (Mean ± SD) Post (%) (Mean ± SD) % Change	96.5 ± 0.2496.87 ± 0.180.38 ± 0.23	96.5 ± 0.2596.9 ± 0.230.43 ± 0.15	1.0000.7780.836
TLC Pre(%) (Mean ± SD) Post (%) (Mean ± SD) % Change	115.63 ± 15.93111.63 ± 13.86−2.96 ± 8.22	111.06 ± 15.14116.00 ± 14.665.42 ± 14.42	0.3130.2040.008
RV Pre(%) (Mean ± SD) Post (%) (Mean ± SD) % Change	208.86 ± 68.23188.16 ± 55.35−6.43 ± 24.9	187.70 ± 68.64207.20 ± 67.6224.89 ± 83.52	0.2770.1470.054
sGaw Pre(%) (Mean ± SD) Post (%) (Mean ± SD) % Change	99.63 ± 51.6297.13 ± 44.24−4.38 ± 76.64	91.23 ± 36.24108.96 ± 61.6919.74 ± 43.22	0.6130.5250.275
RV/TLC (Lt) Pre (Mean ± SD) Post (Mean ± SD) % Change	0.38 ± 0.100.36 ± 0.1010.97 ± 42.01	0.35 ± 0.110.37 ± 0.0911.33 ± 42.68	0.4280.4010.052

Abbreviations: FEV1, forced expiratory volume in 1 second; FVC, forced vital capacity; SpO2, peripheral capillary oxygen saturation; MPIS, modified pulmonary index score; TLC, total lung capacity; RV, residual volume; SGaw, Specific airway conductance.

**Table 4:** Comparison of adverse effects in mesh and jet nebulizer study groups.

	Mesh Group			Jet Group			P*
	Pre n (%)	Post n (%)	P	Pre n (%)	Post n (%)	P	
Palpitation	3(9.7)	10(32.3)	0.016	6(19.4)	8(25.8)	0.500	0.774
Shivering	7(22.6)	9(29.0)	0.500	8(25.8)	11(35.5)	0.375	0.727
Flushing	2(6.5)	1(3.2)	1.000	2(6.5)	4(12.9)	0.625	0.375
Tremor	5(16.1)	7(22.6)	0.125	5(16.1)	8(25.8)	0.250	1.000

* Postmesh vs. Postjet

## 4. Discussion 

To our knowledge, this is the first single blind crossover study in the literature comparing the effects of jet and mesh nebulizers on measures of spirometry and body plethysmography in children with mild/moderate acute asthma. Our findings support the previous evidence found in studies of adults that MN is as effective and safe as JN when used in the treatment of acute asthma in children.

Jet nebulizers are easy to use and inexpensive, so they are commonly used in clinical practice. However, they have a large residual solution volume that cannot nebulize the last of the dose, require an electric source, and cannot be used in a horizontal position. Because of the limitations of JNs, new nebulizers have been developed such as MNs. Compared with JN, MN has high aerosol generation ability and a low residual nebulizer-solution volume, so it can nebulize even µL volumes [7]. There are quite a few studies comparing the efficiency of jet and mesh nebulizers in childhood asthma. 

Adachi et al. studied 73 children with asthma (34 children with mild asthma exacerbation, 39 children in stable condition) who were given a short-acting bronchodilator, procaterol, with conventional and mesh nebulizers. Similar results were observed with both types of nebulizer in terms of the patients’ physical examinations, pulmonary function tests, and side effects. However, the mesh nebulizer was found to have a shorter inhalation time. They concluded that the shorter inhalation time may be an advantage for children who develop a bad temper during inhalation therapy [8]. In another study, pediatric asthma patients aged 8–13 years were randomly divided into 3 groups to compare 3 different mesh nebulizers. Although some differences in lung function improvement had been detected, all 3meshnebulizerswere found to be useful devices in treatingbronchial asthma [9]. A clinical study that compared the clinical utility of the e-Motion mesh nebulizer and a conventional jet nebulizer included patients younger than six years of age with mild asthma attacks. They found no significant difference between clinical scores with 2 devices. Similarly, shortened inhalation time with MN was shown [10]. We did not compare the inhalation time, but we observed similar degrees of improvement in lung functions after beta2-agonist inhalation in both groups. The effectiveness of both nebulizers was comparable. The long-term use of different types of nebulizers might influence the outcome. Dunne and Shortt reported that use of MN was associated with fewer admissions to the hospital, shorter length of stay in the emergency department, and a reduction in total beta2 agonist dose [11]. 

In previous research, WBP was not used to evaluate the effectiveness of MN. WBP measurements are a comparable assessment of lung function to spirometry and provide more detailed information regarding the lung volume. Moreover, WBP offers a more sensitive measure of airway obstruction (sGaw). In our study, the MN group was comparable with the JN group in pretreatment and posttreatment values of TLC, RV, RV/TLC, and sGaw. There was a slight but statistically significant difference between changes in TLC and RV between the groups, mainly due to change in TLC.Its clinical significance requires further investigation. 

Inhaled beta2 agonists have several adverse effects, including palpitation, tachycardia, tremor, and nausea. These effects are dependent upon age, dose, and route of administration. Murayama et al. found an increased heart rate after salbutamol inhalation in both jet and mesh groups for children older than 2 years of age. For the children younger than 2 years, the heart rate increment was not significant. They concluded that the dose of salbutamol solution might be excessive for the older age group [10]. In our study, adverse effects were similar in both nebulizers, but the complaint of palpitation was significantly higher in the MN group. This difference may be related to inhalation efficiency and the dose of the salbutamol. We did not observe heart rate changes before and after inhalation therapy in either group. The doses of salbutamol might need to be reconsidered according to the type of nebulizers. 

The routine administration of the first dose of salbutamol with JN could be a limitation of our study. The patients were experiencing mild-moderate asthma attacks and the JNs were used already in daily practice, so this study design contributed to increased compliance of parents and children. Also, a double-blind study with 2 groups using either JN or MN might add value to the research. 

There are many marketed nebulizers, creating a challenge for clinicians to determine which device is the most suitable for their patients. The therapy choice should be based on the patients’ characteristics and economic status. Many patients use these devices incorrectly, so observation of the patients and proper education is also an important point. Our study supported that MN is as effective and safe as JN in the treatment of acute asthma in children, but further randomized controlled studies are required to guide clinicians in selection of nebulizers.
